# Digital blood in massively parallel CPU/GPU systems for the study of platelet transport

**DOI:** 10.1098/rsfs.2019.0116

**Published:** 2020-12-11

**Authors:** Christos Kotsalos, Jonas Latt, Joel Beny, Bastien Chopard

**Affiliations:** Computer Science Department, University of Geneva, 7 route de Drize, 1227 Carouge, Switzerland

**Keywords:** npFEM, Palabos, GPUs, cellular blood flow, platelet transport

## Abstract

We propose a highly versatile computational framework for the simulation of cellular blood flow focusing on extreme performance without compromising accuracy or complexity. The tool couples the lattice Boltzmann solver Palabos for the simulation of blood plasma, a novel finite-element method (FEM) solver for the resolution of deformable blood cells, and an immersed boundary method for the coupling of the two phases. The design of the tool supports hybrid CPU–GPU executions (fluid, fluid–solid interaction on CPUs, deformable bodies on GPUs), and is non-intrusive, as each of the three components can be replaced in a modular way. The FEM-based kernel for solid dynamics outperforms other FEM solvers and its performance is comparable to state-of-the-art mass–spring systems. We perform an exhaustive performance analysis on Piz Daint at the Swiss National Supercomputing Centre and provide case studies focused on platelet transport, implicitly validating the accuracy of our tool. The tests show that this versatile framework combines unprecedented accuracy with massive performance, rendering it suitable for upcoming exascale architectures.

## 1. Introduction

Blood flow plays an important role in most of the fundamental functions of living organisms. Blood transports oxygen, nutrients, waste products, infectious parasites, tumour cells, to name a few, to tissues and organs. Despite remarkable advances in experimental techniques [1], the type and detail of the information provided remains limited. In the last two decades, computational tools for the direct numerical simulation (DNS) of cellular blood flow have been developed [2]. Except for the fully resolved simulations (the resolution refers to the cellular nature of blood and not to numerical discretization), there is active development on continuum and stochastic models [3,4], which are usually calibrated by the DNS and used for clinically relevant applications (domains of the order of cm^3^) due to their low computational cost compared to the former approach. The DNS complement experiments and have become an essential tool for in-depth investigations. These tools have been used to study various poorly understood phenomena such as the non-Newtonian viscosity of blood [5], thrombus formation [6], the Fåhræus effect [7], the characteristics of the cell free layer [8] and the red blood cell (RBC) enhanced shear-induced diffusion of platelets [4,9]. Apart from physiological conditions, numerical tools have significantly assisted the understanding of pathological conditions [10–12], as they offer a controlled environment for testing a large number of parameters and classifying their effect on blood rheology. With the occurrence of more mature tools, there is an increased focus on developing/analysing lab-on-a-chip systems [13,14] and drug delivery systems [15,16]. Despite such advances, we believe that there is a tremendous space for improvement in terms of fidelity, high performance and clinically relevant scales.

Blood is a complex suspension of RBCs, white blood cells and platelets, submerged in a Newtonian fluid, the plasma. At 35–45% haematocrit, RBCs occupy a substantial volume fraction of blood and have therefore an important impact on blood rheology. The accurate modelling of the collective transport of cells in plasma is of paramount importance, since it can help decipher various *in vivo* and *in vitro* phenomena. A single cubic millimetre of blood (almost a blood drop) contains a few million RBCs, a few hundred thousand platelets and a few thousand white blood cells. Thus, it is extremely challenging to develop a tool capable of simulating blood at cellular level for clinically relevant applications, using high-fidelity models and using a reasonably limited amount of computational resources and time. By clinically relevant applications, our focus is on medical devices (e.g Impact-R platelet function analyser [17], lab-on-a-chip systems) that handle a few mm^3^ of treated blood and study events that fully develop in a few seconds. Indeed, with the current and near future hardware/software, it would be unrealistic to focus on domains of the order of cm^3^ (e.g. vessels) and on events that evolve over several minutes.

The absence of a complete cellular blood flow computational tool constitutes the motivation behind the suggested framework. A complete framework should fulfil a number of criteria, such as generality, robustness, accuracy, performance and modularity. The criteria of generality, robustness and accuracy are addressed in our first description of the tool proposed in Kotsalos *et al.* [18]. In this work, we finalize the framework by introducing an integrated environment that obeys all the above-mentioned criteria, i.e. we introduce a novel GPU implementation of the finite-element method (FEM)-based solid solver and thoroughly present the high-performance computing (HPC) design of the framework, especially the intricate communication patterns between the various modules and processes. The focal point of this study is the computational aspect of the framework and the design decisions taken for an efficient, versatile and modular HPC tool. Our framework is tailored for the fastest supercomputers (namely hybrid CPU/GPU clusters, which are well represented at the high end of the list TOP500 (https://www.top500.org) but also for CPU-only machines) and it is ready to be hosted in upcoming exascale machines. Moreover, the suggested tool, even if it uses state-of-the-art numerical techniques, is not monolithically built upon them: the structural solver for the blood constituents can easily be replaced by another one, such as one based on mass–spring systems. Similarly, the lattice Boltzmann flow solver could be replaced by another option, which however needs to be similarly parallelizable through domain decomposition and allow interaction with solid particles through an immersed boundary method. In the last decade, many groups have been working on HPC-capable cellular blood flow tools [13,19–23] dealing with problems of increased complexity, which do however not reach the goal of a computational framework that fulfils simultaneously all the above-mentioned criteria.

Our team (Palabos development group [24,25]) has developed a numerical method and an HPC software tool for *ab initio* modelling of blood (freely available and integrated in Palabos library, npFEM specialized module). The framework models blood cells like RBCs and platelets individually, including their detailed nonlinear viscoelastic properties and the complex interaction between them. The project is particularly challenging because the large number of blood constituents (up to billions) stands in contrast with the high computational requirement of individual elements. While classical approaches address this challenge through simplified structural modelling of deformable RBCs (e.g. through mass–spring systems) [5,13,26–28], the present framework guarantees accurate physics and desirable numerical properties through a fully featured FEM model [18]. The required numerical performance is achieved through a hybrid implementation, using CPUs (central processing units) for blood plasma and GPUs (graphics processing units) for blood cells. The developed numerical framework is intended to grow to be a general-purpose tool for first-principles investigation of blood properties and to provide an integrative and scalable HPC framework for the simulation of blood flow across scales.

The present work is organized as follows: in Methods, we present the structure of our computational tool and the underlying computational modules; in Results and discussion, we provide a performance analysis on the Piz Daint supercomputer and various case studies on platelet transport.

## 2. Methods

The development of such a solver requires a multi-physics and multi-scale approach as it involves fluid and solid components that may be optimized through a description at different temporal and spatial scales. In order to ensure flexibility and efficiency, we propose a tool based on a modular approach in which code performance and re-usability are central issues. In accordance with the ideas and developments proposed in MMSF (Multi-scale Modelling and Simulation Framework) [29–31], our cellular blood flow computational tool is built on a fluid solver, and a deformable solid bodies solver, whose implementation is potentially left to the preference of the scientists. Here, however, we propose a specific choice based on Palabos flow solver and our deformable bodies npFEM solver. The coupling of the two solvers is realized through a separate interface, as illustrated later, which handles all the needed communication. Note that this coupling interface acts independently of the details of the fluid and solid solvers. It only requires data representing physical quantities which are computed by the two solvers, thus ensuring the independence with respect to the chosen numerical methods.

### 2.1. Computational modules

The understanding and deciphering of a complex transport phenomenon like the movement of platelets requires the deployment of high-fidelity DNS tools that resolve the cellular nature of blood. Platelets are submerged in blood plasma and collide continuously with RBCs that are present in much larger quantities. To capture accurately their trajectories and understand the driving mechanisms behind their motion, we propose a modular and generic HPC framework capable of resolving three-dimensional cellular blood flow simulations. The computational tool is built upon three modules, namely the fluid solver (blood plasma resolution), the solid solver (blood cells) and the fluid–solid interaction (FSI).

The fluid solver is based on the lattice Boltzmann method (LBM) and solves indirectly the weakly compressible Navier–Stokes equations. The three-dimensional computational domain is discretized into a regular grid with spacing Δ*x* in all directions. For this study, we use the D3Q19 stencil, with the Bhatnagar–Gross–Krook (BGK) collision operator and non-dimensional relaxation time *τ* = 2 (higher *τ* gives higher Δ*t*). The time step is determined through the formula $\nu =C_{s}^{2}(\tau -1/2)\Delta t$, where $C_{s}=\Delta x/(\Delta t\sqrt{3})$ is the fluid speed of sound and *ν* is the blood plasma kinematic viscosity. Furthermore, external forcing terms (like the FSI force *f*_imm_) can be incorporated in the LBM through a modification of the collision operator using the Shan–Chen forcing scheme [32]. More information on LBM can be found in [33–35].

The solid solver is based on the recently introduced nodal projective finite elements method (npFEM) by Kotsalos *et al.* [18], which offers an alternative way of describing elasticity. The npFEM framework is a mass-lumped linear FE solver that resolves both the trajectories and deformations of blood cells with high accuracy. The solver has the capability of capturing the rich and nonlinear viscoelastic behaviour of RBCs as shown and validated in [18]. Platelets are simulated as nearly rigid bodies by modifying the stiffness of the material. The implicit nature of the npFEM solver renders it capable of resolving extreme deformations with unconditional stability for arbitrary time steps. Even though the solver is based on FEM and an implicit integration scheme, its performance is very close to the widely used mass–spring systems [5,27], outperforming them in robustness and accuracy [18]. Regarding the blood cell viscoelastic behaviour, the solver uses a two-way approach to handle the response of a cell to the imposed loads over time (Rayleigh and position-based dynamics damping [18]). It should be pointed out that the interior fluid of the cell is implicitly taken into account, as its incompressibility contributes to the potential energy of the body and its viscosity augments the viscosity of the membrane.

The FSI is realized by the immersed boundary method (IBM) and more specifically by the multi-direct forcing scheme proposed by Ota *et al.* [36] (with minor modifications, see electronic supplementary material). The IBM imposes a no-slip boundary condition, so that each point of the surface and the ambient fluid moves with the same velocity. The advantage of the IBM is that the fluid solver does not have to be modified except for the addition of a forcing term *f*_imm_. Moreover, the deformable body and its discrete representation do not need to conform to the discrete fluid mesh, which leads to a very efficient fluid–solid coupling. The exchange of information, between the fluid mesh and the Lagrangian points of the discrete bodies, is realized through interpolation kernels with finite support. The *ϕ*_4_ kernel [37] is used throughout the simulations of the current study. The IBM is an iterative algorithm where the force estimate on a Lagrangian point is computed by the difference of the vertex velocity (coming from npFEM) and the velocity interpolated by the surrounding fluid nodes. Then, this force is spread onto the fluid nodes (*f*_imm_) surrounding the Lagrangian point and the correction affects the collision operator of the LBM. This interpolation between the membranes and the fluid is repeated for a finite amount of steps. For the simulations shown in this article, just one iteration suffices for the required accuracy.

A brief but more instructive overview of the methods presented above can be found in the electronic supplementary material.

### 2.2. Towards stable and robust fluid–solid interaction

There exist two main ways to realize FSI, the monolithic and modular respectively. The former describes the fluid and solid phases through one system of equations and both are solved with a single solver, using the same discretization. Examples include tools that use dissipative particle dynamics to resolve both the fluid and solid. An advantage of the monolithic approach is the consistency of the coupling scheme, which leads to more numerically stable solutions. The main disadvantage is that a single solver potentially falls short of satisfactorily addressing all the physics present in a complex phenomenon. In the modular approach, there is the freedom to choose well optimized solvers to address the different phases. However, the consistent coupling of the solvers becomes a major challenge, especially when the discretization (space and time) is non-conforming. Particularly, our computational framework uses different spatial and time resolutions for the fluid and solid phases, e.g. the solid solver is executed every two iterations (at least), which could potentially introduce coupling instabilities. Instabilities arise mainly from under-resolution and from integration schemes that do not conserve energy and momenta (linear/angular) exactly, thus leading to spirally increasing energy oscillations between the solvers. The remedies suggested below are tailored to the specific framework, but could potentially give useful hints for other implementations.

The IBM requires a match between the lattice spacing Δ*x* and the average edge length $\bar{l}$ of the discretized membranes (triangulated surfaces). The value of the mesh ratio $\bar{l}/\Delta x$ appears to play a minor role as long as it is comprised in the range [0.5, 1.8] [35]. An RBC discretized with 258 surface vertices exhibits a ratio $\bar{l}/\Delta x∼1.6$ with a lattice spacing of 0.5 μm. For low shear rates, this requirement can be further relaxed.

An accurate evaluation of the external forces acting on the immersed boundaries plays a critical role to achieve a consistent coupling. For higher accuracy, we use the hydrodynamic stress tensor ***σ*** projected onto the surface normals instead of the native force term produced by the IBM. Furthermore, compatible with the aim to disregard the interior fluid of blood cells, we found out that the most stable force evaluation scheme comes from measuring ***σ*** at the exterior most distant site from the Lagrangian point contained within the interpolation kernel. Once the force **F**_ext_ is computed, a median filtering in the one-ring neighbourhood of every Lagrangian point attenuates force spikes that could result in energy oscillations.

A meticulous handling of the near-contact regions is deemed highly critical to suppress instabilities. Our approach is to pseudo-resolve these regions through a collision framework (similar to the Morse potential in [12]), and not by refining the involved meshes [8], as the latter approach would lead to impractical numerical simulations due to the high computational cost. The first step of our procedure consists of searching for Lagrangian points belonging to bodies other than the investigated one, that are contained within the interpolation kernel of the current point. If there are no ‘foreign’ particles in the kernel, then no modification is needed. It is then assumed that the interaction physics is appropriately resolved by the fluid field in between bodies. Otherwise, the collision framework takes over, since the evaluation of **F**_ext_ is ‘contaminated’ by the interior fluid of a neighbouring body. Subsequently, the forces on the Lagrangian point from the fluid are disregarded and a collision force, coming from a quadratic potential energy [18], is used instead. The threshold to deactivate the forces from the fluid can be either the boundaries of the interpolation kernel or user specified according to the case. This technique is named by us particle in kernel (PIK) and resolves very accurately colliding bodies (more in electronic supplementary material). We would like to highlight that the actual IBM algorithm is not affected by the PIK technique, but only the evaluation of **F**_ext_. Certainly, the PIK technique has limitations for very high haematocrit in combination with coarse meshes, where the majority of the interpolation kernels could possibly become ‘contaminated’ by foreign particles, i.e. the motion of many membrane vertices will be determined by the collision force, rather than by the stress imparted from the surrounding fluid. In our follow-up research project [38] (combining both *in vitro* and *in silico* experiments), we performed all cellular simulations with and without the PIK technique, and we conclude that, up to 35% haematocrit (higher haematocrit needs further investigation), lattice spacing 0.5 μm, and blood cell resolution as in this study, the PIK technique does not affect the resolved physics (in an order-of-magnitude sense).

The selected IBM version [36] starts from an estimate of a force on the Lagrangian points required to enforce a no-slip condition. This force is spread into the neighbourhood of the points to communicate the constraint of a solid boundary to the fluid. The force estimate is proportional to the difference of the vertex velocity (as computed by the npFEM solver) and the velocity interpolated by the surrounding fluid nodes. The component that can be controlled in the above procedure is the npFEM vertex velocity which, if it exceeds a value U*_max_, then it is truncated towards this threshold. The constant U*_max_ can be comprised between [0.03, 0.1] [34] and is related to the fact that the simulated Mach number (*Ma*) should be ≪ 1, since LBM errors increase dramatically at high *Ma* (= *u*_max_/*C* where *u*_max_ is the maximum simulated velocity in the flow and *C* = Δ*x*/Δ*t* is the lattice speed). This velocity capping proves to be very stabilizing when necessary. If the classic IBM [39] is used instead, then a force capping has the aforementioned stabilizing effect.

Particular attention is needed on the order of execution of the various computational modules (namely fluid, solid and FSI), because time lags between solvers can amplify instabilities. The exact module order of our solver can be found in [18].

### 2.3. High-performance computing design

Direct numerical simulations of cellular blood flow are pushing the computational limits of any modern supercomputer, given the complexity of the underlying phenomena. The amount of unknowns per second varies from millions to trillions [13], and the proposed computational framework is built with genericity, modularity and performance in mind, able to tackle problems in the whole range of unknowns. This computational tool is orchestrated by Palabos [24,25], which is responsible for data decomposition and for the communication layer. Palabos (for **Pa**rallel **La**ttice **Bo**ltzmann **S**olver) is an open-source library for general-purpose computational fluid dynamics, with a ‘kernel’ based on the lattice Boltzmann method. Palabos is written in C++ with extensive use of Message Passing Interface (MPI) and with proven HPC capability, particularly in the domain of computational biomedicine [40–42]. On top of the Palabos core library, we have developed the npFEM solver, which is written in C++ and CUDA (a general-purpose parallel computing platform and programming model for NVIDIA GPUs), and it is derived from the open-source library ShapeOp [43]. There are two active branches of the npFEM library, a CPU-only implementation and a full GPU implementation leveraging NVIDIA GPUs. The GPU parallelization strategy is based on the idea of using one CUDA-thread per Lagrangian point and one CUDA-block per blood cell. This is feasible since the most refined blood cell model has fewer points (discretized membrane) than the maximum allowed number of threads per block (hardware constraint). Keeping all points of a cell within a CUDA-block allows us to compute the entire solver time step in one CUDA-kernel call, and make good use of cache and shared memory [44].

**Figure 1.**
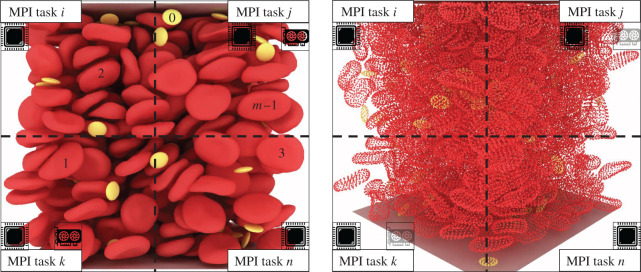
Load balancing of the fluid and solid phases for a modern CPU/GPU computing system. Cubic domain at 35% haematocrit with RBCs and platelets. The immersed bodies exist both as plain Lagrangian points (positions and velocities for the IBM) and augmented Lagrangian points (positions, velocities and other properties relevant only to the solid solver). The LBM and IBM are executed on CPUs, while the npFEM on GPUs (hybrid version of the framework). In most cases, the number of GPUs is much smaller than the available MPI tasks; see Piz Daint with a ratio 1 : 12.

**Figure 2.**
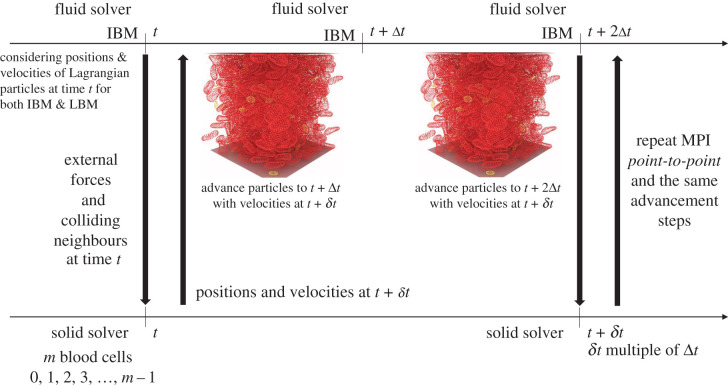
Modular structure of our computational framework. We present the two independent solver streams and the required MPI point-to-point communication for advancing the physical system in time. The decoupling of the solvers leads to a framework that is agnostic to the underlying numerical methods. This diagram remains the same for both the CPU-only version of the framework and the CPU/GPU implementation (hybrid).

Load balancing plays a critical role and impacts the efficiency and scalability of HPC applications. For our hybrid CPU/GPU system, three components require special attention. The first is the representation of the fluid domain through a homogeneous grid. The lattice sites are partitioned by Palabos and are distributed to the available MPI tasks (LBM on CPUs). The second component of the system are the plain Lagrangian points that describe the immersed blood cells for the IBM (see figure 1, right-hand side image). These points are attached to their immediate fluid cells, and thus their dispatching to the available MPI tasks is aligned with the fluid decomposition (IBM on CPUs). The immersed bodies have a dual nature, i.e. they are seen not only as a set of plain Lagrangian points for the IBM but also as a set of augmented Lagrangian particles on the solid solver side (connectivity and material properties on top of position and velocity), see figure 1 both images, where both the plain Lagrangian points and the surfaces are represented. This means that the Lagrangian points are duplicated for the IBM and the npFEM modules. Finally, the MPI tasks that are linked with a GPU are responsible for the solid phase. Blood cells are distributed evenly and statically to the available GPUs in a manner that is disconnected from the attribution of the fluid cells and Lagrangian points to the CPUs (npFEM on GPUs). For example, let us assume that MPI task *j* (see figure 1, left-hand side image) handles *m* blood cells. The blood cell with tag 1 is spatially located in the domain managed by MPI task *k*. Thus, the communication of the external forces, the colliding neighbours and the new state of the body at *t*+1 is realized through MPI point-to-point communication for all surface vertices of the cell. The same parallel strategy is adopted in the CPU-only version. This strategy may seem counterintuitive, leading to a substantial communication load, especially compared to an approach in which the structural solver is attributed to nodes dynamically and retains a processor locality with the coupled flow portions. However, the theoretical scalability of our approach is compatible with the massively parallel vision of the project, as the total amount of communicated data scales with the number of blood cells, and it is independent of the number of computational nodes (except in cases with very few nodes). Indeed every surface vertex is involved in exactly two point-to-point exchanges (a fluid-to-solid and a solid-to-fluid exchange). This fact avoids an over-saturation of the network in a situation of weak scaling, provided that the capacity of the network scales with the number of used compute nodes. It further guarantees that our framework can be connected to any structural solver, as the data provided to the structural solver is always fully contained on a compute node. Our approach further ensures a targeted communication strategy, as the data actually needed by the solver can be handpicked. By providing fully decoupled solvers, we favour a generic and modular approach over ad hoc and monolithic solutions. Figure 2 presents the decoupled structure of our framework, the communication layer and the advancement rules.

## 3. Results and discussion

The goal of this section is to prove the capability of our computational framework to efficiently handle problems of varying size. This is done through an exhaustive presentation of performance metrics that are realized at Piz Daint, the flagship system of the Swiss National Supercomputing Centre (CSCS), ranked sixth worldwide and first in Europe according to the list TOP500 (November 2019). This supercomputer has 5704 GPU nodes equipped with 12 cores and one NVIDIA GPU each, and 1813 CPU nodes equipped with 36 cores each. A complete presentation of the supercomputer can be found in the electronic supplementary material. Our focus is the hybrid version of the framework, where the deformable blood cells are resolved on GPUs, while the blood plasma and the FSI are resolved on CPUs.

For every case study performed in this work, the flow field has a constant shear rate 100 s^−1^, realized with a moving top wall and a fixed bottom wall. This low shear rate is chosen in order to reproduce the experiments in Chopard *et al.* [17] (experiments using the Impact-R platelet function analyser, which generates a Couette flow), and is not due to a computational limitation. Table 1 summarizes all the different domains, represented through their dimension in *x* × *y* × *z* format. The flow direction is parallel to the *z*-axis, the height of the channel spans along the *y*-direction, and periodic boundaries are applied in *x*- and *z*-directions. Furthermore, the haematocrit of the systems varies between 35% and 45%, covering the whole physiological range. The domain is initialized by randomly positioning blood cells (without avoiding interpenetration) and then executing the computational framework for a few thousand steps while the fluid and the FSI solvers are deactivated. This novel cell packing approach is based on the very efficient collision detection offered by Palabos and the unconditional stability of the npFEM solver, which can resolve extreme deformations and interpenetrations. Platelets are simulated as nearly rigid oblate ellipsoids with diameter 3.6 μm, thickness 1.1 μm and volume 6.8 fl, which is an average value for non-activated platelets. The platelet to RBC ratio is 1 : 5, and therefore substantially larger than the physiological one (1 : 10 to 1 : 20 [4]). This is a deliberate choice intended to provide more samples for the statistical analysis of the platelet transport, without affecting the physics. As for the shape of RBCs, the normal biconcave shape is used. The fluid/solid resolutions remain fixed for all case studies, favouring coarser representations for computational efficiency. A complete list of all parameters used in this study can be found in the electronic supplementary material. The performance metrics are followed by an analysis on platelet transport. A series of numerical experiments with varying RBC viscoelasticity and channel height presents the idiosyncratic behaviour of platelets and their sensitivity to various flow factors, while the validity of our simulations is cross-checked (with *in vitro* counterparts and similar *in silico* studies) in an order-of-magnitude sense. This implicit validation increases our confidence in the simulated physics of our solver.

**Table 1.** Numbers of blood cells, RBCs and platelets (PLTs), for different case studies. The *y*-direction is bounded by walls, while the *x*- and *z*-directions are periodic (simulation of Couette flow).

computational domain (μm^3^)	50 × 50 × 50		50 × 100 × 50		50 × 500 × 50		50 × 1000 × 50		100 × 1000 × 100	
PLTs : RBCs = 1 : 5	RBCs	PLTs	RBCs	PLTs	RBCs	PLTs	RBCs	PLTs	RBCs	PLTs
haematocrit 35%	476	95	953	190	4765	953	9531	1906	38126	7625
haematocrit 45%	612	122	1225	245	6127	1225	12255	2451	49020	9804

### 3.1. Performance analysis

Simulations at the spatial scale of millimetres commonly ignore the detailed particulate nature of blood because of the tremendous computational cost, and instead model the effect of particles through continuum modelling. On the other hand, in publications of state-of-the-art fully resolved whole blood simulations [5,16,42,45], the overall spatial scale of the simulation remains very small, of the order of a few tens of micrometres. The suggested HPC framework is built towards the direction of simulating macroscopic flows, the order of mm^3^ of whole blood, while representing the details of the microscopic physics, thus offering users the possibility to address a large range of problems with clinical relevance. In the context of the current scientific goals, the performance metrics of this HPC framework must be considered in the light of weak scaling. Indeed, the purpose of seeking more powerful computational resources is not to improve the resolution or increase the time span of the simulation, but to extend the physical volume of the blood considered in the model.

**Figure 3.**
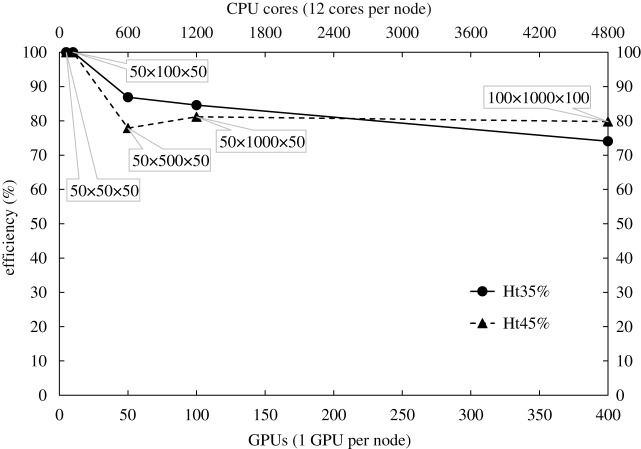
Weak scaling efficiency for various domains at different haematocrit. To better understand the problem sizes consult table 1. The efficiency corresponds to the hybrid version of the code. The GPU nodes in Piz Daint are equipped with 12 cores and one NVIDIA GPU each.

**Figure 4.**
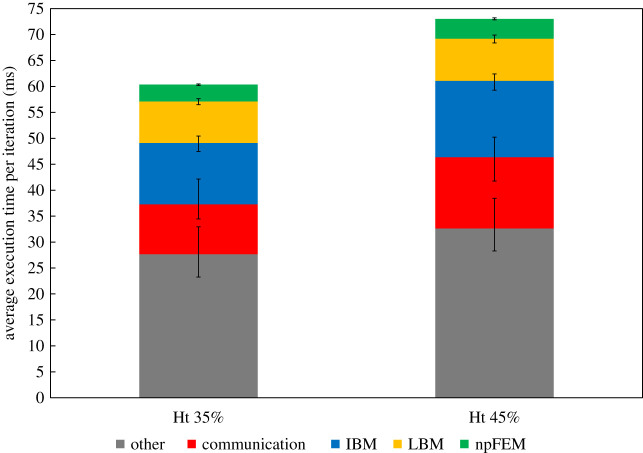
Execution time per iteration for different haematocrit (hybrid version). The ‘other’ contains operations (CPU-only) such as computation of external forces, collision detection, particle advancement and various book-keeping.

**Figure 5.**
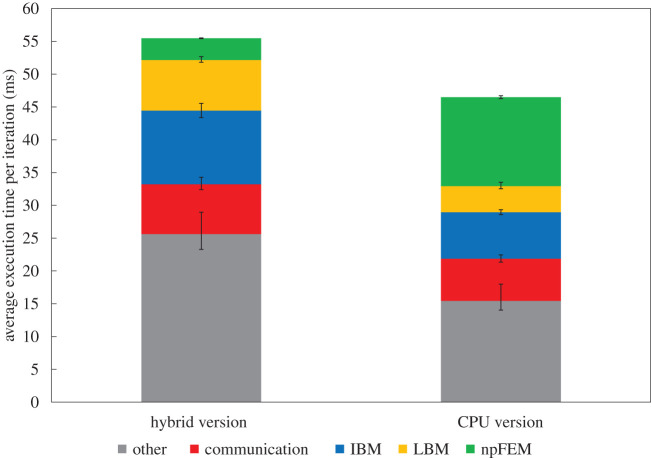
Comparison of execution time per iteration for the hybrid (CPU/GPU) and CPU-only versions of the framework at 35% haematocrit. The GPU nodes have 12 cores and one GPU each, while the CPU nodes have 36 cores each.

In the weak scaling, the computational load per processor (either CPU or GPU) remains constant. Thus, the problem size increases proportionally with the number of computational hardware units. The reference case study is a 50 × 50 × 50 μm^3^ domain, solved on five GPU nodes (*N*_0_) with reference time noted as $t_{N_{0}}$. The weak scaling efficiency is given by $t_{N_{0}}/t_{N}$, where *t*_*N*_ is the time spent in *N* GPU nodes for a domain *N*/*N*_0_ times larger than the reference one. Figure 3 presents the weak scaling efficiency of the proposed computational framework (hybrid version) for a problem growth up to 80 times compared to the reference domain (at 400 GPU nodes). Even if the largest tested domain is still distinctly smaller than 1 mm^3^, the direction of interest (wall-bounded direction) approaches scales of macroscopic extent, and the remaining directions are adequately resolved through periodicity. Our long-term vision for macroscopic flows includes assigning further blood cells per GPU. This on its own requires strong CPU performance to cope with annex preparatory operations (the ‘other’ section on figures 4 and 5), which might be matched by novel, upcoming supercomputing systems. The code presents good efficiency and its performance does not degrade for higher haematocrit. Other frameworks that are based on a modular approach for the coupling of fluid and solid solvers [19,40,42,46] demonstrate an efficiency between 70 and 80%. On the contrary, frameworks that follow the monolithic paradigm [13] deliver a more impressive efficiency, often above 90%. Nevertheless, this is a small penalty to be paid for genericity and modularity over ad hoc solutions.

Figure 4 presents the average execution time per iteration for different haematocrit levels and refers to the hybrid version. The bottom layer of the bars labelled as ‘other’ contains operations (executed on CPUs only) such as computation of external forces, collision detection, particle advancement and various book-keeping. The ‘error’ bars delimit the deviation from the average, where the minimum corresponds to the reference case study and the maximum to the largest case study (table 1) in the context of weak scaling. A striking observation is that the GPU-ported npFEM solver constitutes only approximately 6% of the total execution time, especially if compared with other state-of-the-art implementations [40,41] which report a contribution of the solid solver to over 50% of the overall time. On the other hand, the fluid solver (collide and stream operations of LBM) and the FSI account for about 30% of the execution time with a consistent scaling over larger domains and higher haematocrit. The communication varies around 12–20% of the execution time but does not seem to constitute a bottleneck since it is realized through optimized non-blocking point-to-point communication. The communication refers only to the MPI part, while the CPU/GPU communication is integrated on the npFEM solver, since it constitutes a very small amount of the total execution time. This is firstly because of data locality (MPI task and GPU belong to the same node) and secondly due to the relatively small amount of RBCs per GPU. The ‘other’ group of operations occupy a large portion of the total time, and this hot-spot reflects the choice of genericity and modularity. A possible conclusion from these observations could be to port the remaining parts of the solver to GPUs given the great performance of the solid solver. It is however debatable if this choice would be optimal, given that modern supercomputers tend to become ‘fatter’ in both CPUs and GPUs, as shown in the example of *Summit* with two CPUs per node (21 cores each) and six NVIDIA Volta GPUs, ranked first according to the list TOP500 (November 2019). Thus, the best strategy is to fully exploit the available hardware and not develop one-sided applications. Another counter-argument is that some numerical methods such as the IBM have a large and complex memory footprint that renders them less GPU friendly. An earlier attempt [44] to port the whole framework on GPUs could not serve as a justification to move in this direction with the main bottleneck being the irregular memory patterns of the IBM, even if all computations were performed in just one GPU (data locality advantage).

Figure 5 presents the execution time per iteration for the hybrid (CPU/GPU) version and the CPU-only version. These results come from a weak scaling study for domains up to 50 × 500 × 50 μm^3^ and 35% haematocrit. Figure 5 assumes one-to-one correspondence between GPU and CPU nodes of Piz Daint, e.g. solving the computational domain 50 × 500 × 50 μm^3^ in 50 GPU nodes (one GPU and 12 cores each) for the hybrid version and in 50 CPU nodes (36 cores each) for the CPU-only version. The npFEM solver on its own exhibits a speedup of about 4, favouring the GPU implementation. Moreover, in the CPU-only version it is obvious that the solid solver constitutes an overwhelming part of the overall performance, while in the hybrid version the GPU-porting solves this problem in a very efficient manner. We would like to highlight that in the hybrid version only the npFEM is ported on GPUs while all the other parts are CPU-only. Table 2 presents the energy needed to execute both versions for a fixed number of iterations, as given by the output of Piz Daint. The comparison shows that GPU nodes are more energy efficient than CPU nodes by 16% on average. In cases where blood cells of higher resolution are used (e.g. RBCs discretized at greater than 500 surface vertices), both execution time and energy efficiency of the hybrid version are superior to the CPU-only version (not shown in this study since we focus on coarser representations), and this is due to the better capacity of the GPU-ported part to deal with an increased load. This can be seen from the fact that in the CPU version there are three times more cores than in the hybrid, but the CPU-ported parts are not three times faster (sub-optimal scaling). To summarize, this study does not favour any version of the framework but shows that both are viable and can be efficiently applied, depending on the characteristics of the available hardware.

**Table 2.** Energy to execute 16 000 time steps (2 ms physical time) for both hybrid (GPU nodes) and CPU-only (CPU nodes) versions on Piz Daint for 35% haematocrit.

computational domain (μm^3^)	50 × 50 × 50		50 × 100 × 50		50 × 500 × 50	
nodes	5		10		50	
	GPU	CPU	GPU	CPU	GPU	CPU
energy (MJ)	0.87	0.99	1.71	2.01	10.26	11.76

More performance analysis results can be found in the electronic supplementary material. The main challenge of the computational tools for the simulation of the particulate nature of blood is to solve systems with a sufficient number of blood cells ( ≫ 1000) for a physically relevant duration (approx. 1 s) in a reasonable time (less than a week) with the smallest possible amount of computational resources. More concretely, given the parameters of the current case studies (see electronic supplementary material), the simulation time step is Δ*t* = 0.125 μs. To simulate 1 s physical time, one needs 8 × 10^6^ time steps. For an average execution time of 60 ms per time step (figure 4), the simulation needs approximately five and a half days. The proposed computational framework achieves the aforementioned goals and can be compared with other state-of-the-art solvers [40–42]. The main novelty is that we are able to achieve this by using high-fidelity models for blood cells which have a richer physical content than simple mass–spring systems. To the best of our knowledge, there is no other computational framework using a fully validated FEM-based solid solver that can achieve these target values.

### 3.2. Platelet transport with varying RBC viscoelasticity

RBC viscoelastic behaviour, a collective term for the contribution of both the membrane and the cytoplasm, is a widely accepted factor with critical impact on health and disease. Pathological alterations in RBC deformability have been associated with various diseases [1] such as malaria, sickle cell anaemia, diabetes, hereditary disorders and chronic obstructive pulmonary disease. Despite its crucial role, RBC viscoelasticity is overlooked in the majority of the computational tools for cellular flows. Here, we study the effect of RBC viscoelasticity on platelet transport and discriminate each case with the use of platelet mean square displacement (MSD) in the wall-bounded direction. The parameter altered is *κ*_damping_ as presented in Kotsalos *et al.* [18], which affects the viscoelastic behaviour of RBCs (the higher the more viscous the RBC). The MSD is defined as 〈(*y*_*i*_(*t*) − *y*_*i*_(*t*_0_))^2^〉, with *y*_*i*_ the position of platelet *i* in the wall-bounded *y*-direction. The averaging spans either over all available platelets, i.e. RBC-rich layer (RBC-RL) and cell free layer (CFL), or only over platelets of the RBC-RL. For the current study, we did not measure the properties of the CFL (lack of experimental data to support our findings) and thus we use for the thickness of the CFL a value taken from the study of Vahidkhah *et al.* [8], which is approximately one platelet diameter (3.6 μm). Nonetheless, the impact of this value on the overall study is negligible. The flow set-up includes a constant shear rate at 100 s^−1^, a domain of size 50^3^ μm^3^ and a haematocrit of 35% (table 1). From the simulations, we track at every time step the positions of platelets, and we sample the output at every 10 ms to extract further information, like the MSD or the average distance of platelets from the walls.

**Figure 6.**
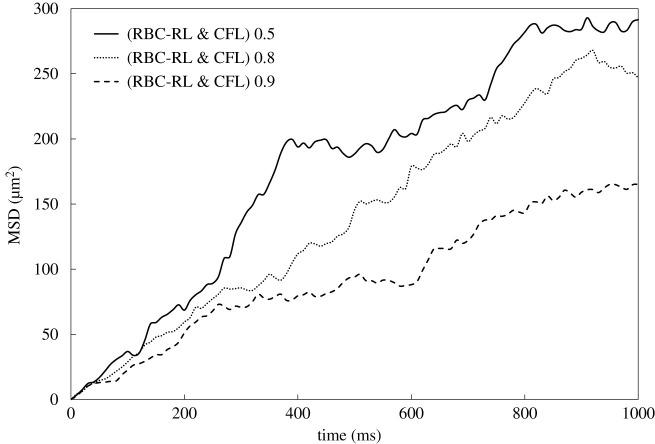
MSD with averaging over all available platelets. The curves correspond to different values of *κ*_damping_ = {0.5, 0.8, 0.9}. The higher the viscoelasticity (*κ*_damping_), the slower the response to the imposed external loads.

Figure 6 presents the MSD over all platelets of the domain for three different values of the RBC viscoelastic parameter *κ*_damping_. There is a clear distinction between the less viscous RBCs (*κ*_damping_ = 0.5) and the more viscous (*κ*_damping_ = 0.9) and their projected effect on platelet transport is apparent on the MSD graphs.

**Figure 7.**
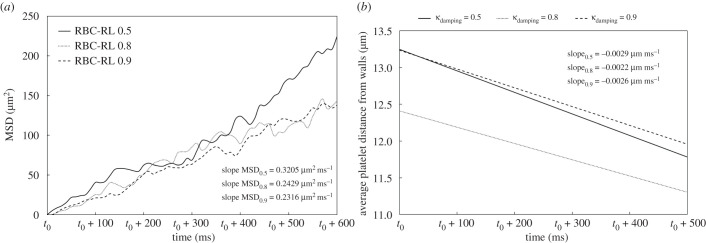
(*a*) MSD with averaging over platelets in the RBC-RL. The slopes at the bottom right are obtained from a linear fitting on each curve. The *t*_0_ corresponds to 300 ms from the beginning of the simulations. (*b*) Average distance of platelets from the walls. As expected from the *in vitro* experiments (Couette flow [17]), the platelets move towards the walls. The curves result from linear fitting on the data extracted every 10 ms.

The computation of the diffusion coefficient (*D*) of platelets demands the MSD to be averaged over platelets of the RBC-RL, and its slope corresponds traditionally to 2*D*. Figure 7*a* presents the MSD and a linear fitting on the curves (see slopes). The diffusion coefficient in all cases is about $O(10^{-10})\;{\text{m}}^{2}\;{\text{s}}^{-1}$, in agreement with previously reported values [8,47,48], thus implicitly validating the accuracy of the tool. It is two to three orders of magnitude higher than the Brownian diffusivity, suggesting RBC-augmented diffusion. An interesting observation in conjunction with the study of Kumar and Graham [3,49], assuming that the more viscous RBCs are more ‘rigid’ (slower response to external forcing), is that the less viscous RBCs *κ*_damping_ = 0.5 lead to higher platelet diffusivity and thus faster concentration towards the walls. The varying diffusivity can be explained by the fact that in heterogeneous collisions the net displacement of the stiff particle (platelet) is substantially larger than that of the floppy particle (RBC) and the displacement is larger for larger rigidity ratio [49]. Thus for less viscous RBCs we expect higher displacements of platelets and thus larger diffusion coefficient, as shown in figure 7. Platelets that reach the walls tend to stay in the CFL and behave as being trapped in this layer. Figure 7 presents as well the average distance of platelets from the walls over time, proving that platelets move towards the walls. It should be noted that there is an agreement between the slopes of MSD and the average distance from walls, i.e. the less viscous RBCs present larger slopes than the more viscous.

### 3.3. Platelet transport for larger geometries

**Figure 8.**
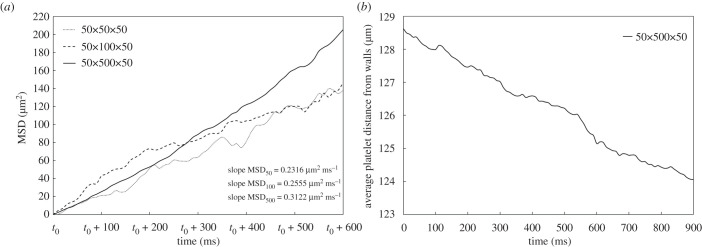
(*a*) Mean square displacement measured in the RBC-rich layer. The *t*_0_ corresponds to 300 ms from the beginning of the simulations. The linear fitting on the MSD gives a slope that is traditionally linked to 2*D*, where *D* is the diffusion coefficient of platelets. (*b*) Average distance of platelets from the walls, showing that platelets move towards the walls.

**Figure 9.**
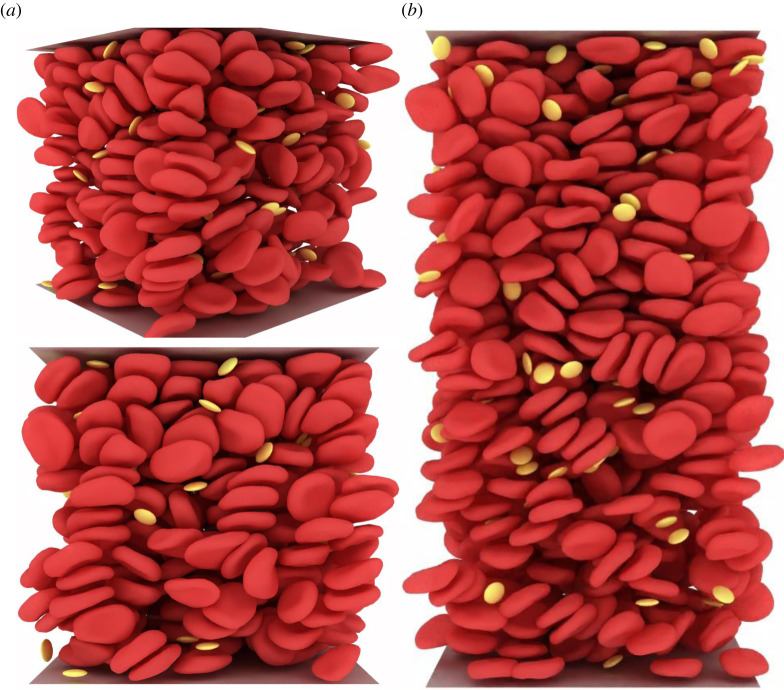
Shear flow generated by our computational framework for fully resolved blood flow simulations. (*a*) Two different viewpoints of the 50^3^ μm^3^ domain at 35% haematocrit. (*b*) A domain 50 × 100 × 50 μm^3^ at 35% haematocrit.

Most studies are bounded by domains of a few micrometres and low haematocrit due to the high computational cost. Nevertheless, interesting phenomena can amplify as sizes increase [50]. Given our HPC-capable framework, we are interested in quantifying the diffusivity of platelets as the channel height varies. Here, a flow field with constant shear rate 100 s^−1^ and 35% haematocrit is considered. The wall-bounded direction takes three different sizes H={50,100,500} μm, while the other periodic directions remain at 50 μm (see table 1). The dimensionless numbers that describe the dynamics of the problem are the capsule Reynolds number $Re_{\mathrm{capsule}}=\dot{\gamma }r^{2}/\nu$, with $\dot{\gamma }$ the shear rate and *r* the characteristic length of the capsule, and the capillary number $Ca=\mu \dot{\gamma }r/B_{\mathrm{Skalak}}$, with *μ* the dynamic viscosity of blood plasma and *B*_Skalak_ the membrane shear modulus (see electronic supplementary material and [18]). Figure 8 shows the mean square displacement in the RBC-RL, and the average platelet distance from the walls, qualitatively validating experimental findings on platelet transport and deposition [17]. The diffusion coefficients for all the different experiments are about two to three orders of magnitude higher than the Brownian diffusivity [8,47], while the increasing diffusivity with the problem size (see slopes in figure 8) is an indication of platelet anomalous diffusion. For a thorough analysis on platelet anomalous diffusion, the reader could consult our follow-up research project [38]. Figure 9 summarizes some of the simulations conducted for the varying channel case study.

## 4. Conclusion

In this study, we provided a computational framework for digital blood, freely available under the Palabos library. The full resolution of the particulate nature of blood is a challenging venture, especially when it is compiled into a framework that is based on generality, modularity and performance without compromising robustness and accuracy. The individual numerical techniques used for the simulation of blood constituents (LBM for the fluid and FEM for the solid phase) are characterized by their high fidelity for capturing physical phenomena, and their coupling has shown to sufficiently resolve the complex interaction between the blood cells.

This kind of computational tool complements the toolset for a digital laboratory. More precisely, the present project complements another research activity based on a coarse-grained approximation of blood using stochastic methods and random walks. The fully resolved models, apart from providing in-depth investigations on various case studies, are used to fine-tune the coarse-grained models, e.g. providing diffusion coefficients of various particles, thus constituting a critical component in this integrative approach towards digital blood. Our future scientific endeavours will be moving to this multi-scale direction as recently depicted in [51].

## Supplementary Material

Digital Blood in Massively Parallel CPU/GPU Systems for the Study of Platelet Transport: Supplementary Material

## Data accessibility

This article has no additional data. The results can be reproduced by the open-source library Palabos and its specialized module for cellular blood flow simulations (Palabos-npFEM, https://palabos.unige.ch/).
