# Supplementary material for: Digital blood in massively parallel CPU/GPU systems for the study of platelet transport
Source: Interface Focus. 2020 Dec 11;11(1):20190116. doi: 10.1098/rsfs.2019.0116 (PMC7739916; doi:10.1098/rsfs.2019.0116)
Supplement: Digital Blood in Massively Parallel CPU/GPU Systems for the Study of Platelet Transport: Supplementary Material [file rsfs20190116supp1.pdf]

Digital Blood in Massively Parallel CPU/GPU Systems for the Study of Platelet Transport

Supplementary Material

Publication in Interface Focus, The Royal Society

Christos Kotsalos*[1], Jonas Latt[1], Joel Beny[1], and Bastien Chopard[1]

[1]Computer Science Department, University of Geneva, 7 route de Drize, CH-1227 Carouge, Switzerland

---

Abstract

We propose a highly versatile computational framework for the simulation of cellular blood flow focusing on extreme performance without compromising accuracy or complexity. The tool couples the lattice Boltzmann solver Palabos for the simulation of blood plasma, a novel finite element method (FEM) solver for the resolution of deformable blood cells, and an immersed boundary method for the coupling of the two phases. The design of the tool supports hybrid CPU-GPU executions (fluid, fluid-solid interaction on CPUs, deformable bodies on GPUs), and is non-intrusive, as each of the three components can be replaced in a modular way. The FEM-based kernel for solid dynamics outperforms other FEM solvers and its performance is comparable to state-of-the-art mass-spring systems. We perform an exhaustive performance analysis on Piz Daint at the Swiss National Supercomputing Centre and provide case studies focused on platelet transport, implicitly validating the accuracy of our tool. The tests show that this versatile framework combines unprecedented accuracy with massive performance, rendering it suitable for upcoming exascale architectures.

Keywords: npFEM, Palabos, GPUs, Cellular Blood Flow, Platelet Transport

---

*Corresponding author: christos.kotsalos@unige.ch

Simulation Parameters

Parameter	Value
Δx	$0.5\ \mu m$
relaxation time τ	2
Δt	$\nu = C_s^2(\tau - 1/2)\Delta t$
Skalak B,C,D	$5, 5000, 35\ pN/\mu m$
$k_{bending}$	$1\ pN\mu m$
k_{volume}	250
Rayleigh α_D, β_D	$0, 0.01$
κ_{damping}	$\{0.5, 0.7, 0.8, 0.9\}$
IBM cycles	1
RBC mesh resolution (surface vertices)	258
Platelet mesh resolution (surface vertices)	66
Fluid density, kinematic viscosity	$1025\ fg/\mu m^3, 1\ \mu m^2/\mu s$

Methods

The computational framework used in this study simulates the particulate nature of blood, i.e. blood cells that are submerged in blood plasma. In more details, we suggest a high-performance computational framework for fully resolved 3D blood flow simulations. The tool is based on three computational modules, namely the fluid solver, the solid solver and the fluid-solid interaction (FSI).

The fluid solver is based on the lattice Boltzmann method (LBM) [1] and the module uses Palabos [2, 3] as for the implementation of LBM. Palabos stands for Parallel Lattice Boltzmann Solver and it is an open source software maintained by the Scientific and Parallel Computing Group (SPC) at the University of Geneva. The FSI is done via an immersed boundary method known as multidirect forcing scheme and its implementation is realised in Palabos. Lastly, the solid solver is based on the nodal projective finite elements method (npFEM) [4] and the module uses the npFEM library developed and maintained by Palabos team, based on the open-source ShapeOp library [5]. The npFEM library is integrated in Palabos core library.

In this section, a basic and brief outline of the used methods is given for the sake of completeness of the article. For a thorough presentation of the numerical methods, the interested readers should consult Kotsalos *et al.* [4].

Nodal Projective FEM (npFEM)

The deformable blood cells are simulated by the nodal projective finite elements method. The npFEM is a mass-lumped linear FE solver that resolves both the trajectories and deformations of blood cells with high accuracy. The solver has the capability of capturing the rich and non-linear viscoelastic behaviour of red blood cells as shown and validated in [4]. The rest of blood cells, platelets for the current study, are simulated as nearly-rigid bodies by increasing the stiffness of the material in the solid solver.

Let us assume a surface mesh consisting of n vertices with positions $\boldsymbol{x} \in \mathbb{R}^{n\times 3}$ and velocities $\boldsymbol{v} \in \mathbb{R}^{n\times 3}$. The evolution of the body (trajectory & deformed shape) in time follows Newton's laws of motion. At time t_n, the system is described by $\{\boldsymbol{x}_n, \boldsymbol{v}_n\}$. The external forces are defined as $\boldsymbol{F}_{ext} \in \mathbb{R}^{n\times 3}$ and are due to the fluid-solid interaction, while the internal forces are $\boldsymbol{F}_{int} \in \mathbb{R}^{n\times 3}$ and are due to the material properties. The internal forces are given by $\boldsymbol{F}_{int}(\boldsymbol{x}) = -\sum_i \nabla E_i(\boldsymbol{x})$, where $E_i(\boldsymbol{x})$ is a scalar discrete elemental potential energy (the summation of all elemental potential energies results in the total elastic potential energy of the body). The *implicit Euler time integration* leads to the following advancement rules:

$$\boldsymbol{v}_{n+1} = \frac{\boldsymbol{x}_{n+1} - \boldsymbol{x}_n}{h}, \tag{1}$$

$$\boldsymbol{F}_{int}(\boldsymbol{x}_{n+1}) + \boldsymbol{F}_{ext}(\boldsymbol{x}_n) - \mathbf{C}\boldsymbol{v}_{n+1} = \mathbf{M}\frac{\boldsymbol{v}_{n+1} - \boldsymbol{v}_n}{h}, \tag{2}$$

where $\mathbf{M} \in \mathbb{R}^{n\times n}$ is the mass matrix, h is the time step, subscripts n and $n+1$ refer to time t and $t+h$, respectively and $\mathbf{C} \in \mathbb{R}^{n\times n}$ is the damping matrix acting like a proxy for viscoelasticity. Except for Rayleigh damping, we augment the viscoelastic behaviour through another proxy called position-based dynamics damping [6] (more details in [4]). The mass matrix is built by lumping the total mass of the body (including the interior fluid) on the mesh vertices, resulting in a diagonal structure. We should mention as well that the implicit nature of

the npFEM solver renders it capable of resolving very high deformations with unconditional stability for arbitrary time steps. Other integration schemes, such as the explicit Runge-Kutta, have been tested for even higher accuracy, but since we are interested in investigating the collective behaviour of multiple blood cells, this enhanced accuracy in modelling the trajectory of a single blood cell did not affect the overall behaviour. Equations (1) & (2) can be combined and converted into an optimisation problem as

$$\min_{\boldsymbol{x}_{n+1}} \; g(\boldsymbol{x}_{n+1}) = \frac{1}{2h^2} \left\| \widetilde{\mathbf{M}}^{\frac{1}{2}} (\boldsymbol{x}_{n+1} - \boldsymbol{y}_n) \right\|_F^2 + \sum_i E_i(\boldsymbol{x}_{n+1}), \tag{3}$$

where $\widetilde{\mathbf{M}} = \mathbf{M} + h\mathbf{C}$, $\boldsymbol{y}_n = \boldsymbol{x}_n + h\widetilde{\mathbf{M}}^{-1}\mathbf{M}\boldsymbol{v}_n + h^2\widetilde{\mathbf{M}}^{-1}\boldsymbol{F}_{ext}$ and $||\cdot||_F$ is the Frobenius norm. Indeed, setting the derivative of equation (3) to zero (thereby minimising the objective function), we recover Newton's second law of motion. The solution to the above optimisation problem gives the system's state at the next time step, i.e. $\{\boldsymbol{x}_{n+1}, \boldsymbol{v}_{n+1}\}$ and thus resolves the trajectory and deformed state of the body at time $t+h$. The steps to minimise equation (3) are thoroughly described in Kotsalos *et al.* [4], but roughly a quasi-Newton approach is employed that converges to $\boldsymbol{x}_{n+1}$ through a sequence of iterations ($k = 1, ...$) as

$$\boldsymbol{x}_{n+1}^{k+1} \leftarrow \boldsymbol{x}_{n+1}^{k} - \widetilde{\mathbf{H}} \nabla g(\boldsymbol{x}_{n+1}^{k}), \tag{4}$$

where $\widetilde{\mathbf{H}}$ is an approximation of the Hessian of $g(\boldsymbol{x}_{n+1})$. The Hessian approximation is built on top of the observation that most of the potential energies that describe the behaviour of a red blood cell are of quadratic form [4].

The theory introduced above is not restricted to membranes only, but it can straightforwardly resolve fully 3D bodies, i.e. the ones discretized by tetrahedra. This section gave a broad overview of the topic and the main novelty of this approach is a cleaner way to describe elasticity through a variational point of view.

Lattice Boltzmann Methods (LBM)

The lattice Boltzmann method (LBM) is employed to solve indirectly the Navier-Stokes equations. Virtual particles, also known as populations, move on a regular grid/lattice and collide at the lattice nodes. LBM is a second-order accurate solver for the weakly compressible Navier-Stokes equation, where the weak compressibility refers to errors that amplify as Mach number tends to one [1].

Let us consider a 3D incompressible fluid flow, with density ρ and kinematic viscosity ν. The fluid domain is discretised into a regular grid with spacing Δx in all directions. The fluid is represented as a group of fictitious particles residing at lattice sites and move to the neighbouring nodes along a fixed set of discrete directions with given discrete velocities at discrete time steps Δt [7]. For this study, we use a D3Q19 stencil and the fluid populations at each site are allowed to move to its 19 immediate neighbours with 19 different velocities $\{\boldsymbol{c}_i\}_{i=0}^{18}$. The degrees of freedom of the model are the populations, a group of 19 variables $\{\boldsymbol{f}_i\}_{i=0}^{18}$ on each cell, also know as fluid density distribution functions, each relating to the probable number of fluid particles moving along the ith direction. The evolution of the fluid through the discrete time is realised in two steps, i.e. the collision and streaming. The collision refers to the redistribution of the fluid populations at each lattice site and the streaming to their movement to neighbouring sites. By discretising the Boltzmann equation (which describes the evolution of the continuous distribution function f) in velocity space, physical space and time, we get the lattice Boltzmann equation (LBE)

$$f_i(\boldsymbol{x}, t) \leftarrow f_i(\boldsymbol{x}, t) + \Omega_i(\boldsymbol{x}, t) \qquad \text{(Collision)} \tag{5}$$

$$f_i(\boldsymbol{x} + \boldsymbol{c}_i\Delta t, t + \Delta t) = f_i(\boldsymbol{x}, t) \qquad \text{(Streaming)}, \tag{6}$$

which describes the collision and propagation of the mesoscopic particle packets. In all our simulations we use the linearised single-relaxation-time BGK [8] formulation for Ω_i. The macroscopic properties of the fluid such as density ρ, velocity $\boldsymbol{u}$ and hydrodynamic stress tensor $\boldsymbol{\sigma}$ can directly be recovered at each lattice node and per time step from the moments of the distribution functions f_i. External forcing terms (like the FSI force f_{imm}) can be incorporated in the LBM through a modification of the collision term Ω using the Shan-Chen forcing scheme [9].

Important quantities to consider in LBM are the lattice speed $C = \Delta x/\Delta t$, the relaxation time τ, the fluid speed of sound $C_s = C/\sqrt{3}$ and their coupling through the physical kinematic viscosity ν as

$$\nu = C_s^2(\tau - 1/2)\Delta t. \tag{7}$$

A particular point of interest is that in lattice Boltzmann simulations, if one fixes the spatial discretization and the relaxation time for a given fluid (known viscosity), then there is no freedom over the selection of the time

discretization, as it is dictated by the diffusive scaling formula (7). If Δt is not defined through equation (7), then physics are violated.

It can be proved that the updating rules (with BGK for Ω) (5) recover the incompressible Navier-Stokes to second order in both space and time. Since the LBM is obtained by the linearised expansion of the original kinetic theory-based LB equation, the resulting macroscopic variables converge to the Navier-Stokes equations with order Ma^2 [7], where $\mathrm{Ma} = u_{max}/C$ is the computational Mach number and u_{max} is the maximum simulated velocity in the flow. Therefore for numerical accuracy and stability, it is required that $\mathrm{Ma} \ll 1$.

For a complete overview of the lattice Boltzmann method, the reader should consult the work done by Krüger *et al.* [1, 10] & Feng *et al.* [7].

Immersed Boundary Method (IBM)

The coupling between blood cells (solid phase) and blood plasma (fluid phase) is realised through the immersed boundary method (IBM). Essentially, IBM imposes a no-slip boundary condition, so that each point of the surface and the ambient fluid move with the same velocity. The key idea is that the surface of the deformable object is viewed as a set of Lagrangian points, which do not have to conform with the fluid mesh/lattice. The fluid feels the presence of the body only via a force field f_{imm}. The interaction between the off-lattice marker points (Lagrangian points) and the fluid is done via interpolation stencils, i.e. regularised Dirac delta functions. There are many variations of IBM [11] and in this study we are using a modified version of the multi-direct forcing method introduced by Wang and colleagues [12], in a form close to the one proposed by Ota *et al.* [13].

The goal is to compute a forcing term f_{imm} along the immersed boundaries and apply it as body force to the fluid (Shan-Chen forcing scheme). Let us denote with $\boldsymbol{x}$ and $\boldsymbol{X}_k$ the Eulerian and Lagrangian points respectively, with $\boldsymbol{U}_k$ the velocity of the Lagrangian point k as computed by the solid solver (npFEM) and with $\boldsymbol{u}^*(\boldsymbol{x}, t)$ the fluid velocity as recovered by the distribution functions f_i. Using an interpolation stencil (W), the velocity on the Lagrangian point $\boldsymbol{X}_k$ is given by

$$\boldsymbol{u}^*(\boldsymbol{X}_k, t) = \sum_{\boldsymbol{x}} \boldsymbol{u}^*(\boldsymbol{x}, t) W(\boldsymbol{x} - \boldsymbol{X}_k) \Delta x^3, \quad (8)$$

where the interpolation stencil is the ϕ_4 with support $4\Delta x$ [14].

The body force $\boldsymbol{f}_{imm}$ is determined by the following iterative procedure:

Step 0. Initial estimation of the body force on the Lagrangian points by

$$\boldsymbol{f}_0(\boldsymbol{X}_k, t) = \frac{\boldsymbol{U}_k(t) - \boldsymbol{u}^*(\boldsymbol{X}_k, t)}{\Delta t} A_k, \quad (9)$$

where A_k is the corresponding surface area of the Lagrangian point k.

Step 1. Distribute the force estimate from the Lagrangian point $\boldsymbol{X}_k$ to the neighbouring lattice sites at a range dictated by the interpolation kernel ϕ_4. Correct the fluid velocity as $\boldsymbol{u}^* + \boldsymbol{f}_l \Delta t$ and re-interpolate the corrected velocity at the Lagrangian points.

Step 2. Re-update the body force on Lagrangian point $\boldsymbol{X}_k$

$$\boldsymbol{f}_{l+1}(\boldsymbol{X}_k, t) = \boldsymbol{f}_l(\boldsymbol{X}_k, t) + \frac{\boldsymbol{U}_k(t) - \boldsymbol{u}_l(\boldsymbol{X}_k, t)}{\Delta t} A_k, \quad (10)$$

and repeat from **Step 1**.

The above iterative procedure can be applied for as many cycles as the application demands. For simulations with multiple blood cells, even one cycle gives satisfactory results. The body force computed by the last cycle on the Eulerian grid is the $\boldsymbol{f}_{imm}$.

The immersed body force term $\boldsymbol{f}_{imm}$ could be used as the $\boldsymbol{F}_{ext}$ for the solid solver. However, we make use of the hydrodynamic stress tensor for higher accuracy. To compute the force on the Lagrangian point k, we project the stress tensor $\boldsymbol{\sigma}$ onto the surface normal $\boldsymbol{n}_k$.

We should highlight that in the classic immersed boundary method the same fluid covers the whole computational domain. However, this contradicts with the existence of interior fluid in blood cells, of different viscosity and density, than the exterior blood plasma. Our approach is to completely disregard the interior fluid and view it as an artefact of IBM. To do so, we compute $\boldsymbol{F}_{ext}$ on the solid phase by summing only the points $\boldsymbol{x}$ that are on the outside region of the bodies. The effect of the interior fluid of the blood cells is implicitly taken into account from the npFEM solver by considering both its incompressibility and viscosity as additional terms to the viscoelasticity of the membrane. Thus, the bodies are simulated as entities that encapsulate all the different components of blood cells [4].

Particle In Kernel (PIK) technique

The correct handling of colliding bodies, especially for flows at high hematocrit, is of paramount importance for accuracy and stability reasons. Bodies that approach each other very closely activate the collision framework which has to assist/replace the hydrodynamic interactions because of under-resolution of the latter. Additionally, IBM and the computation of external forces on the immersed bodies make use of interpolation kernels that are drastically affected when they are "contaminated" by "foreign" particles. This means that the interpolated values consider regions that are not well-resolved, e.g. the interior fluid of blood cells, and this could potentially lead to accuracy and stability problems. The Particle In Kernel (PIK) technique is essentially part of the collision framework and makes sure that the correct forces are used at all times. In Figure 1 there is a visual description of PIK for an investigated point $\boldsymbol{x}_i$ (dot) and 2 potential collision candidates (stars). If there is no "foreign" particle, i.e. particle belonging to another body, inside the kernel (sphere with $4\Delta x$ diameter in 3D) then the fluid resolution is sufficient to handle the flow field. Otherwise, we disregard the fluid force and the collision framework gives the force instead. The closest "foreign" particle is chosen to be the colliding neighbour and the desired location of $\boldsymbol{x}_i$ is noted as $\boldsymbol{p}_i$ (projection). Subsequently, a collision potential energy and the corresponding force is introduced in the solid solver. The threshold to disregard the forces from the fluid can be either the boundaries of the interpolation kernel or user specified according to various factors, e.g. boundary conditions, hematocrit, flow field. This approach guarantees to use only non-contaminated interpolation kernels and a clean collision framework. This technique affects only the computation of $\boldsymbol{F}_{ext}$ on the immersed boundaries and has no impact on the IBM steps.

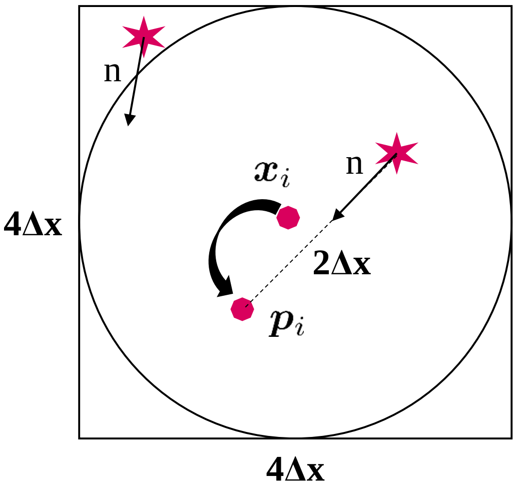

$$E_i = \frac{w_{col}}{2} ||\boldsymbol{p}_i - \boldsymbol{x}_i||_F^2$$

$$F_i = w_{col}(\boldsymbol{p}_i - \boldsymbol{x}_i)$$

Figure 1: Particle In Kernel (PIK) technique and corresponding energy/force pair introduced in the npFEM solver.

Piz Daint @ CSCS

Piz Daint is the current flagship system of the Swiss National Supercomputing Centre (CSCS). This supercomputer is a hybrid Cray XC40/XC50 system. According to the list TOP500 (November 2019), Piz Daint is ranked 6th worldwide, while it is the most powerful system in Europe. The specifications of the supercomputer are the following:

Model	Cray XC40/XC50
XC50 Compute Nodes	Intel Xeon E5-2690 v3 @ 2.60GHz (12 cores, 64GB RAM) and NVIDIA Tesla P100 16GB - 5704 Nodes
XC40 Compute Nodes	Two Intel Xeon E5-2695 v4 @ 2.10GHz (2 x 18 cores, 64/128 GB RAM) - 1813 Nodes
Login Nodes	Intel Xeon CPU E5-2650 v3 @ 2.30GHz (10 cores, 256 GB RAM)
Interconnect Configuration	Aries routing and communications ASIC, and Dragonfly network topology
Scratch capacity	8.8 PB

Running the CUDA "deviceQuery" script on an XC50 node, we get the characteristics of the GPU that it is equipped with (see Figure 5).

Performance Analysis

Goal of this section is to complement the performance analysis of the main article. Figure 2 summarises the weak scaling case studies (we omit the cases at 45% hematocrit). Figure 3 presents the weak scaling comparison between the hybrid version (npFEM on GPUs, LB/IBM on CPUs) and the CPU-only version. Porting part of a library on GPUs can cause extra data management, book-keeping and overhead from new data structures, thus most of the times the efficiency of the ported hybrid library demonstrates a degradation. Obviously (Figure 3), this is not the case for our computational framework where we manage to keep the same performance, keeping the overhead at a minimum level. Strong scaling plays a critical role when there is an abundance of computational power. Also, strong scaling partially indicates the scalability of a tool and thus where applicable it is an interesting metric to show. Figure 4 presents the speedup of the strong scaling for a domain of size 50x1000x50 μm^3. The speedup is given as $\frac{t_{N_0}}{t_N}$, where t_{N_0} is the time spend in N_0 GPU nodes and t_N in N GPU nodes. The speedup is good but not ideal and the reason for this is the extra book-keeping needed for satisfying modularity and genericity of the framework.

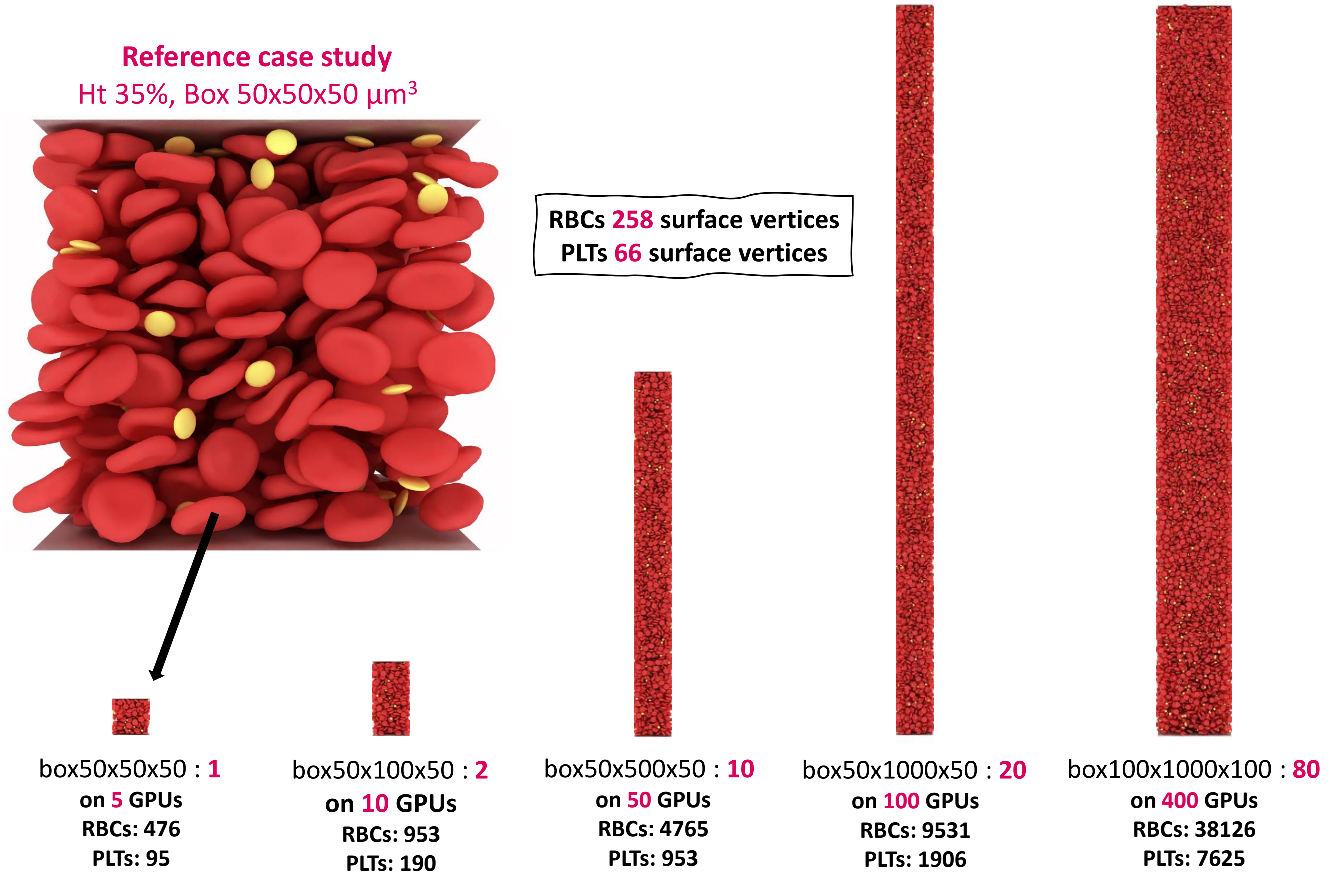

Figure 2: Summary of the weak scaling case studies. For each domain, we present the problem growth in comparison to the reference domain, the number of GPU nodes used to solve the domain and the numbers of blood cells injected in the field. The numbers of blood cells refer to the case of 35% hematocrit.

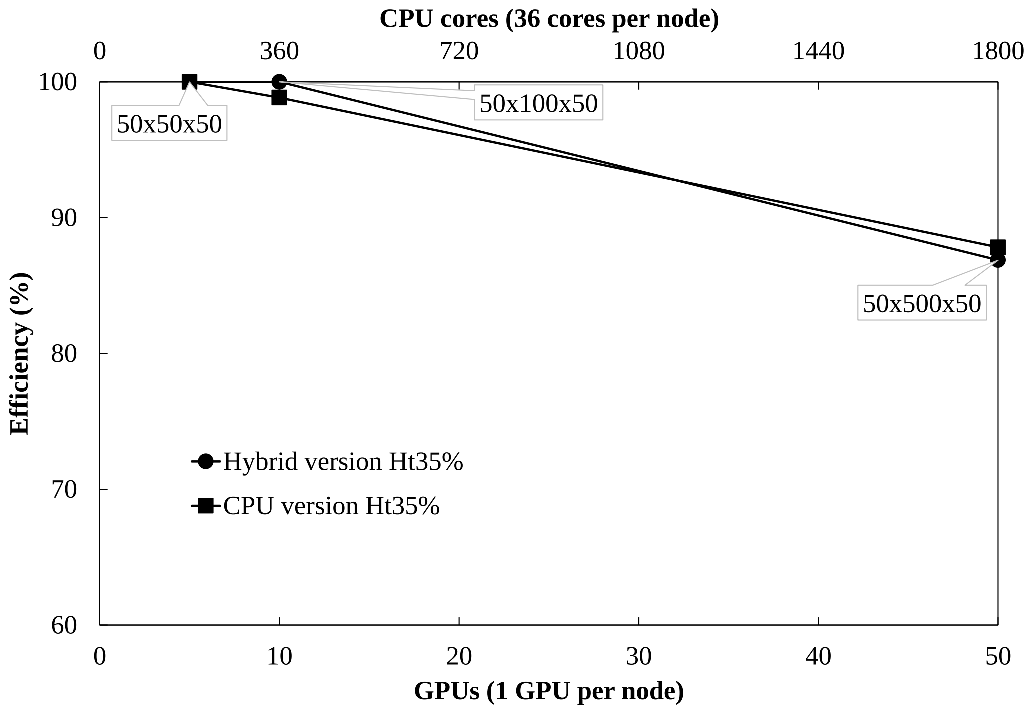

Figure 3: Weak scaling efficiency comparison of the hybrid (CPU/GPU) version with the CPU-only version. The hybrid version is executed on GPU nodes (12 cores and one NVIDIA GPU each), while the CPU-only version on the CPU nodes (36 cores each) of Piz Daint. The upper axis refers to the CPU nodes, while the lower axis refers to the GPU nodes.

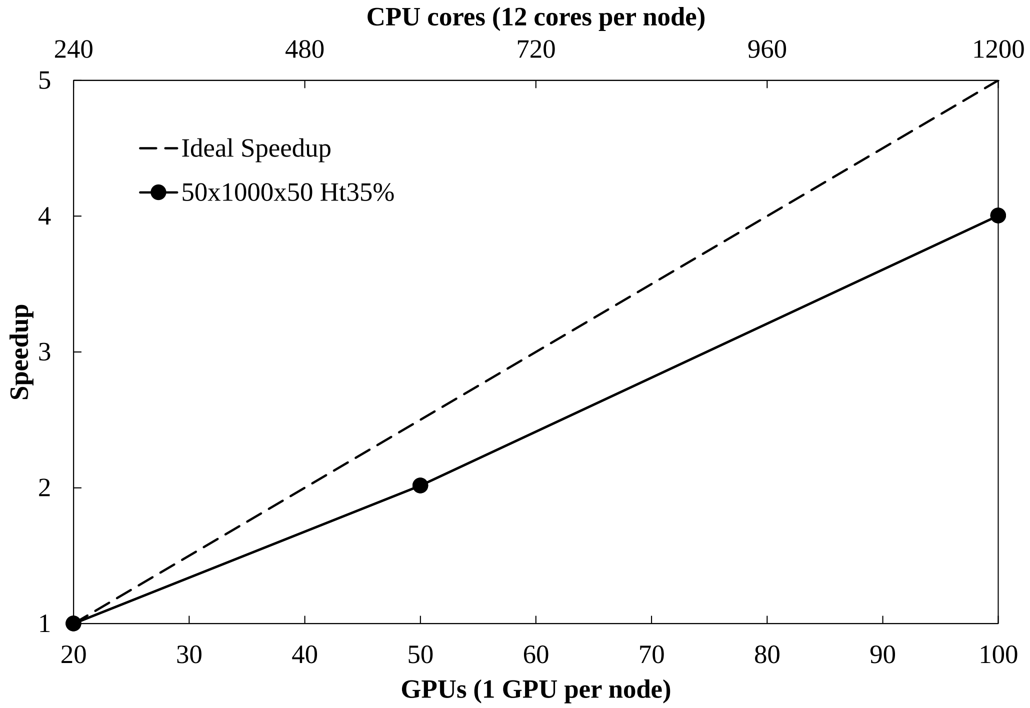

Figure 4: Strong scaling of the hybrid version of the code for a large domain.

```
Detected 1 CUDA Capable device(s)

Device 0: "Tesla P100-PCIE-16GB"
  CUDA Driver Version / Runtime Version          9.2 / 9.1
  CUDA Capability Major/Minor version number:    6.0
  Total amount of global memory:                 16281 MBytes (17071734784 bytes)
  (56) Multiprocessors, ( 64) CUDA Cores/MP:     3584 CUDA Cores
  GPU Max Clock rate:                            1329 MHz (1.33 GHz)
  Memory Clock rate:                             715 Mhz
  Memory Bus Width:                              4096-bit
  L2 Cache Size:                                 4194304 bytes
  Maximum Texture Dimension Size (x,y,z)         1D=(131072), 2D=(131072, 65536),
3D=(16384, 16384, 16384)
  Maximum Layered 1D Texture Size, (num) layers  1D=(32768), 2048 layers
  Maximum Layered 2D Texture Size, (num) layers  2D=(32768, 32768), 2048 layers
  Total amount of constant memory:               65536 bytes
  Total amount of shared memory per block:       49152 bytes
  Total number of registers available per block: 65536
  Warp size:                                     32
  Maximum number of threads per multiprocessor:  2048
  Maximum number of threads per block:           1024
  Max dimension size of a thread block (x,y,z): (1024, 1024, 64)
  Max dimension size of a grid size    (x,y,z): (2147483647, 65535, 65535)
  Maximum memory pitch:                          2147483647 bytes
  Texture alignment:                             512 bytes
  Concurrent copy and kernel execution:          Yes with 2 copy engine(s)
  Run time limit on kernels:                     No
  Integrated GPU sharing Host Memory:            No
  Support host page-locked memory mapping:       Yes
  Alignment requirement for Surfaces:            Yes
  Device has ECC support:                        Enabled
  Device supports Unified Addressing (UVA):      Yes
  Device supports Compute Preemption:            Yes
  Supports Cooperative Kernel Launch:            No
  Supports MultiDevice Co-op Kernel Launch:      No
  Device PCI Domain ID / Bus ID / location ID:   0 / 2 / 0
  Compute Mode:
     < Exclusive Process (many threads in one process is able to use
::cudaSetDevice() with this device) >

deviceQuery, CUDA Driver = CUDART, CUDA Driver Version = 9.2, CUDA Runtime Version =
9.1, NumDevs = 1
Result = PASS
```

Figure 5: “deviceQuery” script on an XC50 node of Piz Daint @ CSCS

References

1. Krüger, T. *et al. The Lattice Boltzmann Method* (2017).
2. Latt, J. *et al.* Palabos: Parallel Lattice Boltzmann Solver. *Computers and Mathematics with Applications.* ISSN: 08981221 (2020).
3. *Palabos* `https://palabos.unige.ch/`. 2019.
4. Kotsalos, C., Latt, J. & Chopard, B. Bridging the computational gap between mesoscopic and continuum modeling of red blood cells for fully resolved blood flow. *Journal of Computational Physics* **398.** ISSN: 0021-9991 (2019).
5. *ShapeOp* `https://www.shapeop.org/`. 2014.
6. Müller, M., Heidelberger, B., Hennix, M. & Ratcliff, J. Position based dynamics. *Journal of Visual Communication and Image Representation* **18,** 109–118 (2007).
7. Feng, Y. T., Han, K. & Owen, D. R. J. Coupled lattice Boltzmann method and discrete element modelling of particle transport in turbulent fluid flows: Computational issues. *International Journal for Numerical Methods in Engineering* **72,** 1111–1134 (2007).
8. Bhatnagar, P. L., Gross, E. P. & Krook, M. A Model for Collision Processes in Gases. Small Amplitude Processes in Charged and Neutral One-Component Systems. *Physical Review* **94,** 511–525 (May 1954).
9. Shan, X. & Chen, H. Lattice Boltzmann model for simulating flows with multiple phases and components. *Physical Review E* **47,** 1815–1819 (Mar. 1993).
10. Krüger, T. *Computer Simulation Study of Collective Phenomena in Dense Suspensions of Red Blood Cells under Shear* (Vieweg+Teubner Verlag, 2012).
11. Mittal, R. & Iaccarino, G. Immersed Boundary Methods. *Annual Review of Fluid Mechanics* **37,** 239–261 (2005).
12. Wang, Z., Fan, J. & Luo, K. Combined multi-direct forcing and immersed boundary method for simulating flows with moving particles. *International Journal of Multiphase Flow* **34,** 283–302 (2008).
13. Ota, K., Suzuki, K. & Inamuro, T. Lift generation by a two-dimensional symmetric flapping wing: Immersed boundary-lattice Boltzmann simulations. *Fluid Dynamics Research* **44** (2012).
14. Mountrakis, L., Lorenz, E. & Hoekstra, A. G. Revisiting the use of the immersed-boundary lattice-Boltzmann method for simulations of suspended particles. *Phys. Rev. E* **96,** 013302 (1 2017).
